# Hyaluronan activated-metabolism phenotype (HAMP) in pancreatic ductal adenocarcinoma

**DOI:** 10.18632/oncotarget.27172

**Published:** 2019-09-24

**Authors:** Yuzan Kudo, Shiro Kohi, Keiji Hirata, Michael Goggins, Norihiro Sato

**Affiliations:** ^1^ Department of Surgery 1, School of Medicine, University of Occupational and Environmental Health, Kitakyushu, Japan; ^2^ Department of Pathology, Sol Goldman Pancreatic Cancer Research Center, Johns Hopkins University School of Medicine, Baltimore, Maryland, USA

**Keywords:** hyaluronan, synthesis, degradation, pancreatic cancer, prognosis

## Abstract

**Background:** The aggressiveness of pancreatic ductal adenocarcinoma (PDAC) is enhanced by its interactions with stromal extracellular matrix, notably with hyaluronan (HA). Our previous studies have demonstrated increased expression of genes involved in HA synthesis and degradation in PDAC, suggesting the presence of an autocrine mechanism which accelerates the production of low-molecular-weight HA.

**Results:** A subset of PDAC (20% of cell lines and 25% of tissues) showed overexpression of multiple genes encoding both HA-synthesizing and HA-degrading enzymes, displaying a phenotype defined as an HA activated-metabolism phenotype (HAMP). Interestingly, HAMP+ cells were more susceptible to the treatment with an HA synthesis inhibitor and HA degradation inhibitor than HAMP- cells. Patients with HAMP+ tumors were significantly associated with shorter survival than those with HAMP- tumors (P = 0.049).

**Methods:** We investigated transcriptional profiling of genes involved in HA synthesis (including *HAS2* and *HAS3*) and degradation (including *HYAL1* and *KIAA1199*) in a panel of PDAC cell lines and primary tissues. Response of PDAC cells to treatment with an HA synthesis inhibitor (4-methylumbelliferone) or HA degradation inhibitor (dextran sulfate) was examined by cell migration assay. Survival was determined by Kaplan–Meier curve and compared by log-rank test.

**Conclusions:** The present study identified a novel phenotype, HAMP, characterized by activation of HA metabolism pathways, in PDAC. HAMP should be further investigated as a prognostic marker as well as a target for personalized medicine.

## INTRODUCTION

Pancreatic ductal adenocarcinoma (PDAC) is one of the most aggressive and lethal cancers worldwide, currently ranking the fourth leading cause of cancer death in Western countries and Japan. In general, PDAC exhibits poor response to chemotherapy or immunotherapy; therefore, the identification of more effective therapy is urgently needed [1]. Recently, personalized medicine (also known as precision medicine) has attracted much interest in the field of cancer therapy. However, there have been only a few targetable molecules/pathways and their corresponding drugs identified in PDAC [2].

Progression of cancer depends largely on tumor microenvironment composed of extracellular matrix (ECM), such as collagen, fibronectin, laminin, and hyaluronan (HA). These ECM components, along with a variety of stromal cells, orchestrate a host stromal response that ultimately supports invasive and metastatic processes of cancer cells [3]. PDAC is characterized by a dense desmoplastic stroma containing a large amount of ECM. Among the ECM components, HA has been shown to abundantly accumulate in PDAC and provide a favorable environment for tumor progression [4–6]. We demonstrated that HA is strongly expressed in primary PDAC tissues, with a staining being detected both in tumor and stromal components [5]. Importantly, strong HA expression was an independent prognostic factor in patients with PDAC undergoing resection [5].

HA is a large polymer, of a molecular weight of ranging from 10^5^ to 10^7^ Da in its naïve form, composed of two monosaccharides, glucuronic acid and N-acetyl-glucosamine [7]. HA is synthesized by hyaluronan synthases localized in the plasma membrane (including HAS1, HAS2, and HAS3) and extruded into the extracellular space [8]. The extracellular HA is incorporated into the cells and degraded into smaller fragments by enzymes including hyaluronidases (such as HYAL1 and HYAL2) [7, 9] and KIAA1199/CEMIP/HYBID [10]. HA is involved in many signaling pathways to regulate a wide variety of cellular processes, including cell adhesion, migration, and proliferation by interacting with specific cell surface receptors [11]. In particular, low-molecular-weight HA, produced upon degradation, is strongly associated with malignant phenotype of various cancers [12–14], suggesting the important role of abnormal HA metabolism in cancer progression.

In many cancer types, two major processes of HA metabolism (synthesis and degradation) have been reported to be activated [15, 16]. Our previous studies have shown increased expression of *HAS2*, *HYAL1*, and *KIAA1199* in PDAC [5, 17, 18]. Furthermore, we have shown that HA, especially low-molecular-weight HA, promotes migratory ability of PDAC cells [14]. These findings led us to hypothesize the existence of an HA activated-metabolism phenotype (HAMP) in which a series of HA metabolism processes are activated to consequently accelerate the production of low-molecular-weight HA.

The aim of the present study was to identify HAMP in PDAC by expression profiling of multiple genes involved in HA metabolism. We also determined the clinical implications of HAMP as a therapeutic target and prognostic marker.

## RESULTS

### Identification of HAMP in PDAC cell lines

**Figure 1:**
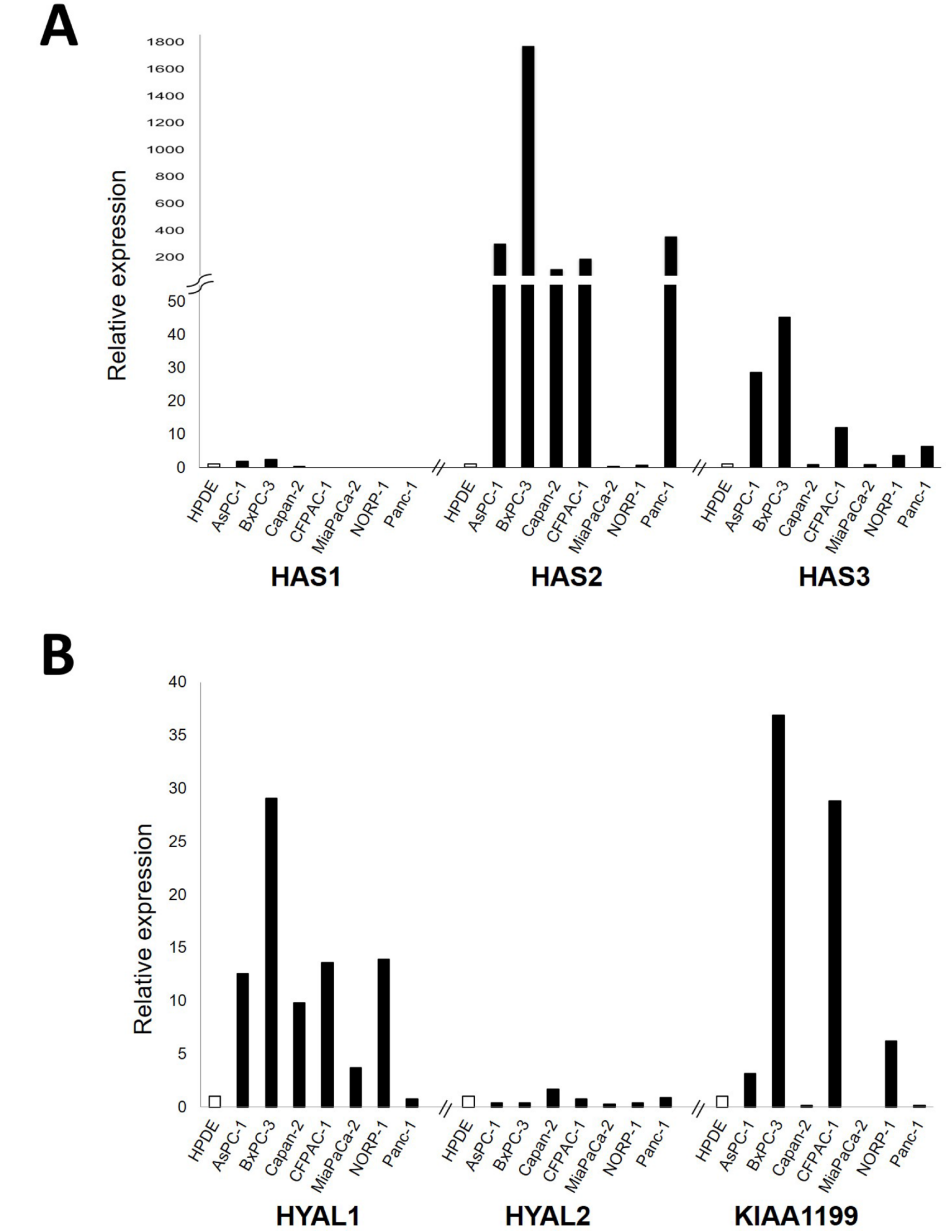
Relative mRNA expression levels of major genes responsible for HA synthesis, including *HAS1*, *HAS2*, and *HAS3* (**A**) and those for HA degradation, including *HYAL1*, *HYAL2*, and *KIAA1199* (**B**) in a panel of 7 PDAC cell lines. Each value was defined when the expression level in HPDE (as a control) was set to 1.

In an attempt to identify genes related to HA metabolism that are highly expressed in PDAC, we first examined mRNA expression levels of major genes responsible for HA synthesis (*HAS1*, *HAS2* and *HAS3*) and degradation (*HYAL1*, *HYAL2*, and *KIAA1199*) in a panel of 7 PDAC cell lines. Of the HA synthesizing enzyme genes, *HAS2* and *HAS3* were overexpressed in a subset of PDAC cell lines as compare to the control cell line HPDE (Figure 1A). Of the HA degrading enzyme genes, *HYAL1* and *KIAA1199* were highly expressed in a subset of PDAC cell lines (Figure 1B).

**Figure 2:**
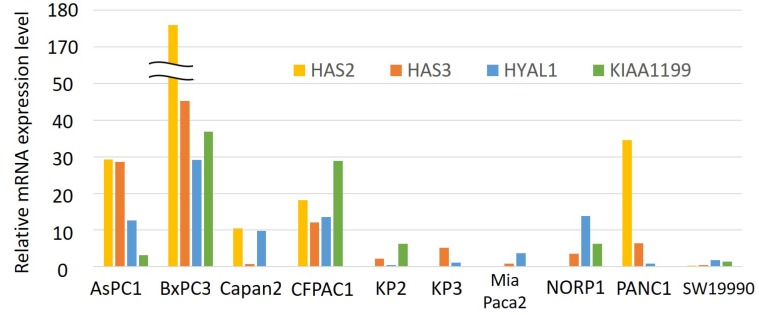
Relative mRNA expression levels of *HAS2*, *HAS3*, *HYAL1*, and *KIAA1199* in a panel of 10 PDAC cell lines. Each value was defined when the expression level in HPDE (as a control) was set to 1. Only HAS2 expression was divided by 10.

We therefore investigated the expression profiling of these four genes (*HAS2*, *HAS3*, *HYAL1*, and *KIAA1199*) in a panel of 10 PDAC cell lines (Figure 2). When we looked at expression of these four genes in each cell line, it seems that some cell lines show increased expression of multiple genes whereas others show low expression of them.

**Figure 3:**
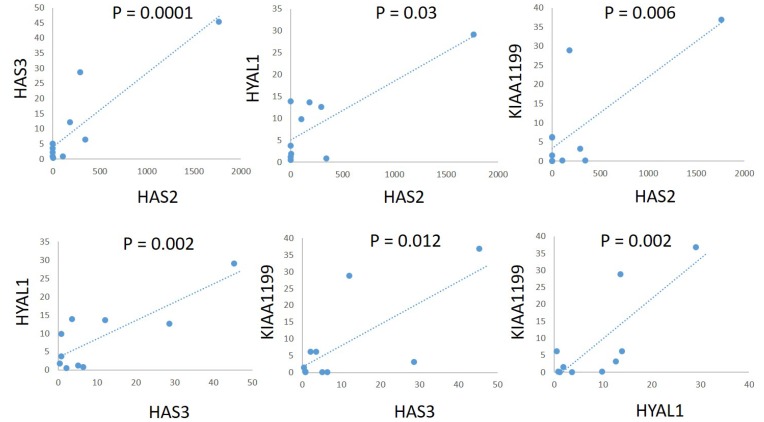
Correlations of mRNA expression levels of *HAS2*, *HAS3*, *HYAL1*, and *KIAA1199* in a panel of PDAC cell lines. There was a significant positive correlation between *HAS2* and *HAS3* (*r* = 0.891, *P* = 0.0001), *HAS2* and *HYAL1* (*r* = 0.803, *P* = 0.003), *HAS2* and *KIAA1199* (*r* = 0.762, *P* = 0.006), *HAS3* and *HYAL1* (*r* = 0.83, *P* = 0.002), *HAS3* and *KIAA1199* (*r* = 0.723, *P* = 0.012), and *HYAL1* and *KIAA1199* (*r* = 0.812, *P* = 0.002).

To explore the correlation between expression of these genes among cell lines, we used Spearman’s rank correlation coefficient to examine the expression levels of two of these genes. There was a significant positive correlation between *HAS2* and *HAS3* (*r* = 0.891, *P* = 0.0001), *HAS2* and *HYAL1* (*r* = 0.803, *P* = 0.003), *HAS2* and *KIAA1199* (*r* = 0.762, *P* = 0.006), *HAS3* and *HYAL1* (*r* = 0.83, *P* = 0.002), *HAS3* and *KIAA1199* (*r* = 0.723, *P* = 0.012), and *HYAL1* and *KIAA1199* (*r* = 0.812, *P* = 0.002) (Figure 3).

**Figure 4:**
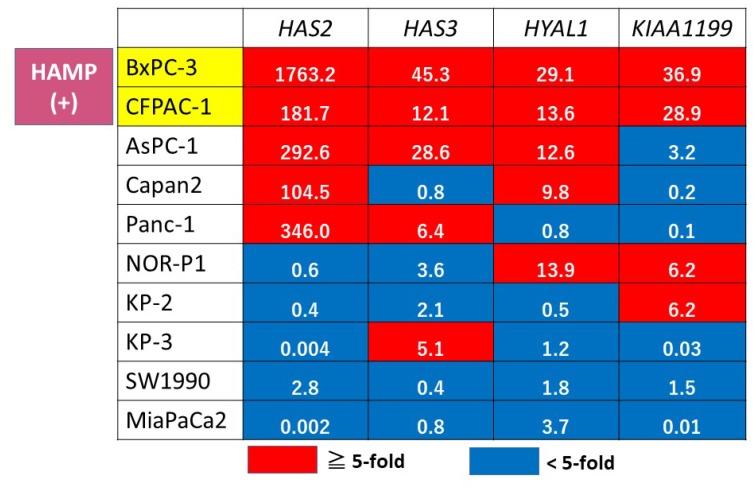
Gene expression profiling of HA metabolism genes in PDAC cell lines. A red box indicates overexpression (> 5-fold relative to HPDE), and blue indicates non-overexpression (< 5-fold). The actual fold change is given in each box. HAMP+ was defined when all of the 4 tested genes were overexpressed.

We then classified the expression pattern of these genes into overexpression (defined as > 5-fold relative to the expression of HPDE) or non-overexpression (< 5-fold) in all PDAC cell lines (Figure 4). This expression profiling identified two cell lines (BxPC3 and CFPAC1) displaying overexpression of all of the four genes tested, which were defined as HAMP.

**Figure 5:**
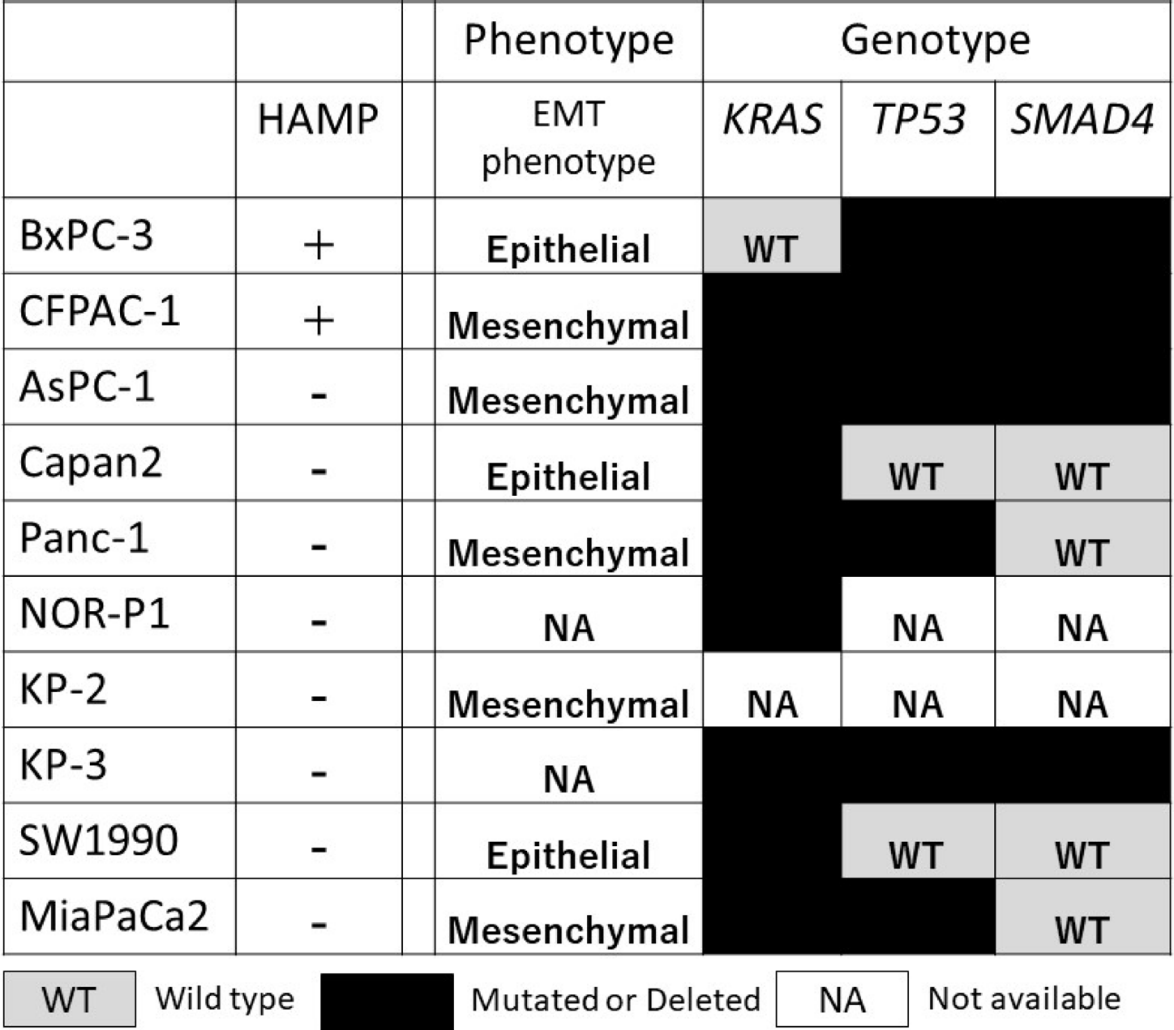
Correlation between HAMP and other known phenotype (epithelial/mesenchymal phenotype) and genotype (genetic alterations in the *KRAS*, *TP53*, and *SMAD4*) in a panel of 10 PDAC cell lines. WT, wild type; black box, mutated or deleted; NA, information not available.

HAMP was not associated with known phenotype (epithelial mesenchymal transition phenotype) and genotype (*KRAS*, *TP53*, and *SMAD4* alterations), possibly representing an independent phenotype (Figure 5).

**Figure 6:**
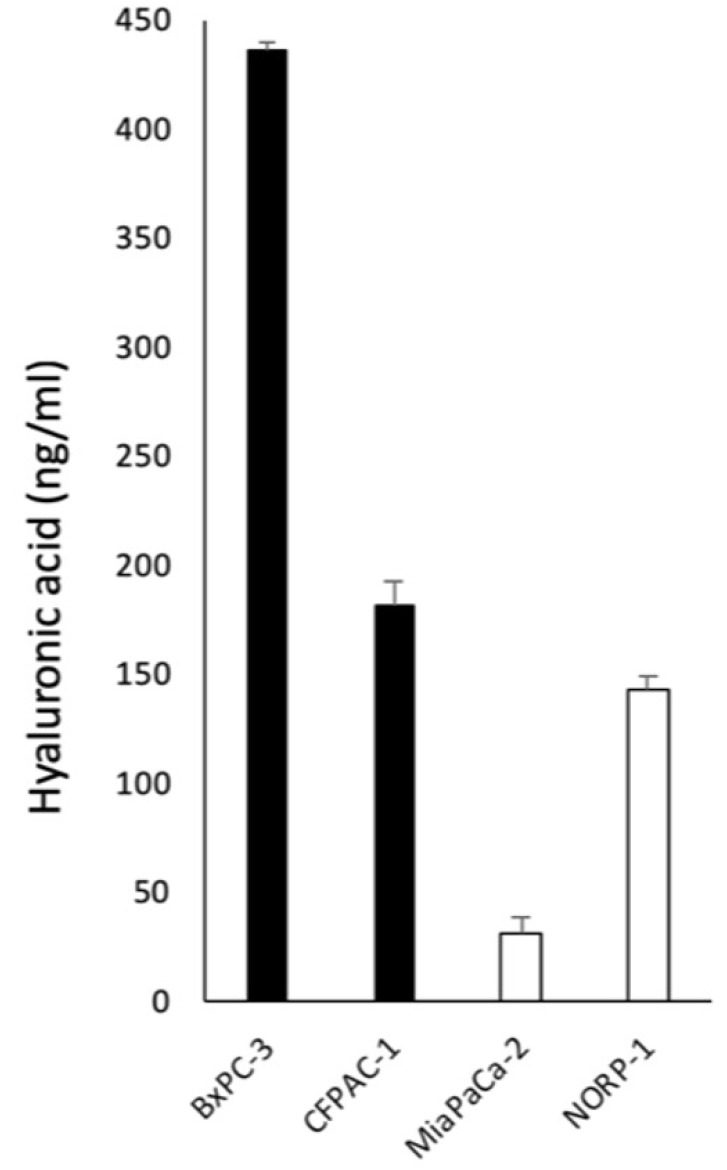
Concentrations of HA in conditioned media from HAMP + cell lines (BxPC3 and CFPAC1) and HAMP- cell lines (MiaPaCa2 and NOR-P1). The HA concentration appeared higher in HAMP+ cell lines than HAMP− cell lines.

To confirm the increased HA metabolism in HAMP at protein level, we examined HA concentration in conditioned media from HAMP+ cell lines (BxPC3 and CFPAC1) and HAMP- cell lines (MiaPaCa2 and NOR-P1). The HA concentration appeared higher in HAMP+ cell lines than HAMP- cell lines (Figure 6).

### Different responses to inhibitors of HA synthesis and hyaluronidase by different HAMP status

**Figure 7:**
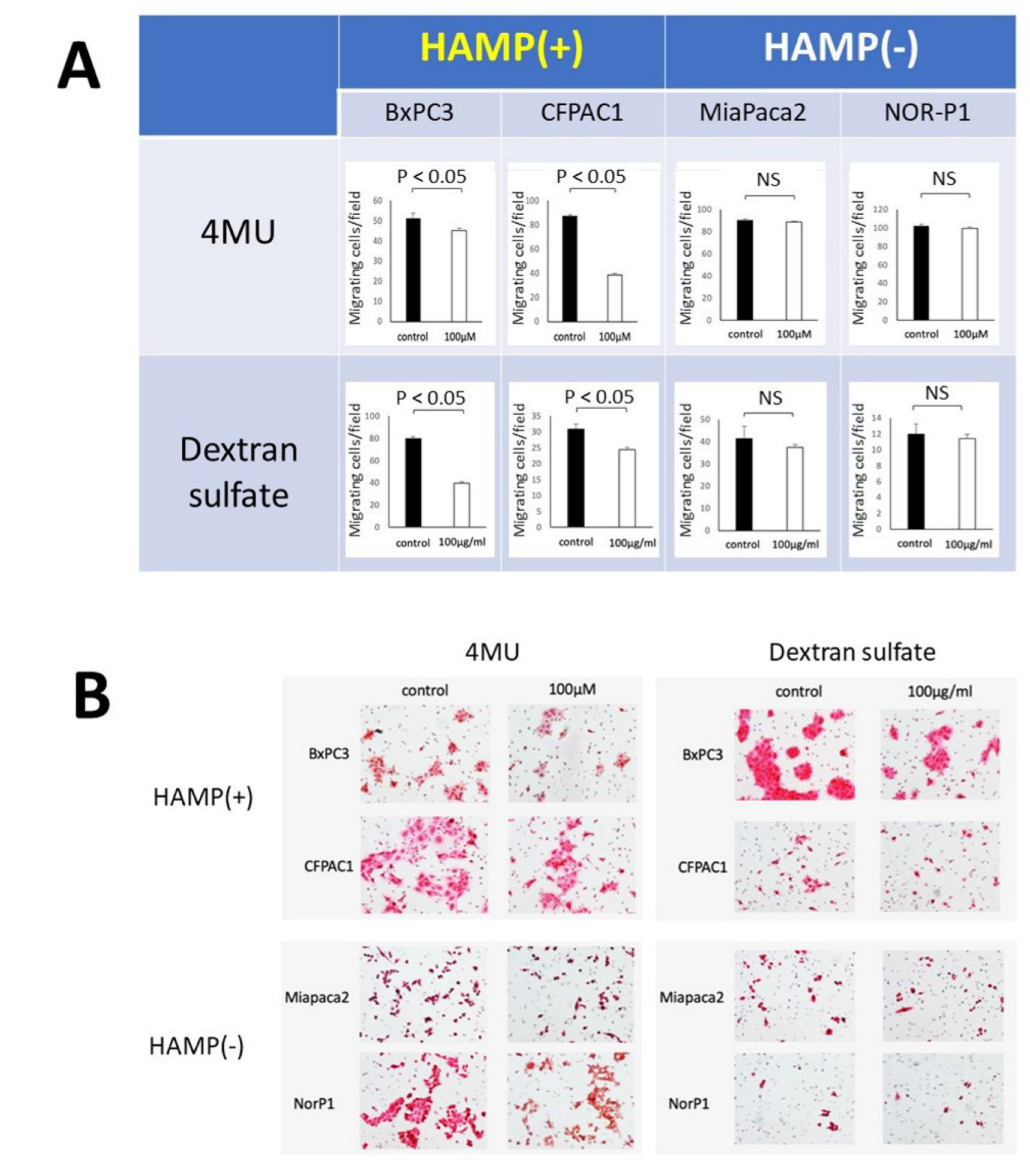
Responses of PDAC cell lines with different HAMP status to inhibitors of HA synthesis (4-MU) or degradation (dextran sulfate). Migrating cells were determined after treatment with 4-MU (100 μM) or dextran sulfate (100 mg/ml) in a transwell assay. (**A**) The number of migrating cells per field after treatment, showing a significant decrease only in two HAMP+ cell lines. (**B**) Representative pictures of migrating cells.

Previous studies have shown that treatment with HA synthesis inhibitor (4-MU) and hyaluronidase inhibitor (dextran sulfate) resulted in decreased HA production and migratory ability in some PDAC cell lines [14, 18]. We hypothesized that HAMP+ cells are more susceptible to inhibitors of HA synthesis or degradation than HAMP- cells. To test this hypothesis, we compared the responses to these treatments between two HAMP+ cell lines (BxPC3 and CFPAC1) and two HAMP- cell lines (MiaPaCa2 and NOR-P1). Treatment with 4-MU (100 μM) resulted in a significant decrease in the number of migrating cells in the HAMP+ cell lines but not in the HAMP- cell lines (Figure 7). Similarly, treatment with dextran sulfate (100 mg/ml) resulted in a significant decrease in the number of migrating cells in the HAMP+ cell lines but not in the HAMP- cell lines (Figure 7).

### Prognostic implication of HAMP in PDAC patients

**Figure 8:**
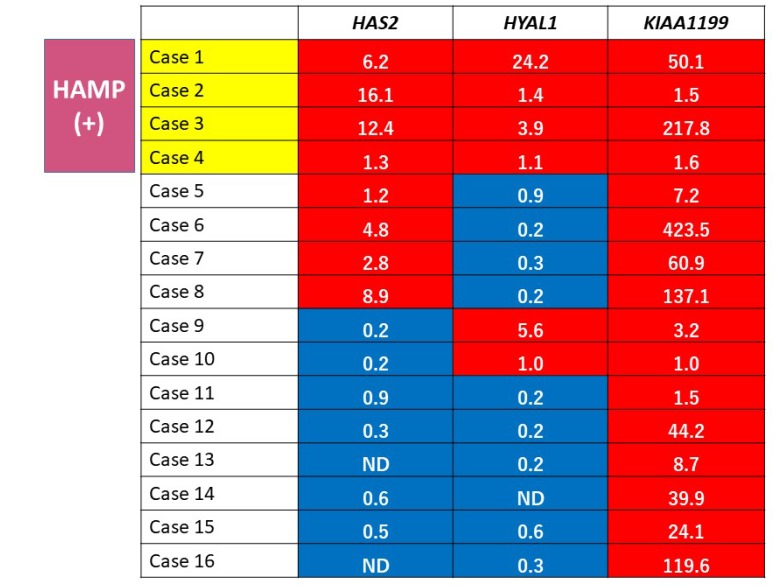
Gene expression profiling of HA metabolism genes in PDAC tissues. A red box indicates increased expression (tumor/non-tumor > 1) and blue box indicates non-increased expression (tumor/non-tumor < 1). The actual fold change is given in each box. HAMP+ was defined when expressions of all of the 3 tested genes were increased.

To explore prognostic relevance of HAMP in PDAC patients, we investigated expression profiling of *HAS2*, *HYAL1*, and *KIAA1199* mRNA in PDAC tissues obtained from 16 patients (7 male and 9 female patients with the average age of 66.7 years) who underwent pancreatectomy (Table 1). Increased expression was defined when the expression level was higher in tumor than in the corresponding non-tumor tissue. Expression profiling identified four cases (25%) showing increased expression of all of the genes tested, which were defied as HAMP+ (Figure 8). There were no significant differences in patient’s backgrounds and clinicopathological variables, including age, sex, serum CEA and CA19-9 level, UICC stage, histological grade (well, moderately, poorly differentiated), lymphatic/vessel/neural invasion, residual tumor, and adjuvant chemotherapy between patients with HAMP+ and those with HAMP- (Table 1).

**Table 1:** Clinicopathological characteristics of 16 PDAC patients with and without HAMP+

Variable		HAMP+ (*n* = 4)	HAMP− (*n* = 12)	Total (*n* = 16)	*P*
Age (years)		58.3 (46–70)	69.5 (33–81)	66.7 (33–81)	0.674
Gender	M	3	4	7	0.146
	F	1	8	9	
serum CEA (ng/mL)		5.75 (2.8–8.4)	5.79 (1–13.6)	5.78 (1–13.6)	0.983
serum CA19.9 (U/mL)		115.8 (15.4–328.3)	156.5 (0.8–989.4)	146.3 (0.8–989.4)	0.712
UICC-T					0.485
	pT1	0	2	2	
	pT2	1	2	3	
	pT3	3	5	8	
	pT4	0	3	3	
UICC-N					0.728
	pN0	1	3	4	
	pN1	3	9	12	
UICC-M					NA
	pM0	4	12	16	
	pM1	0	0	0	
UICC-Stage					0.545
	IA	0	1	1	
	IB	1	1	2	
	IIA	0	0	0	
	IIB	3	7	10	
	III	0	3	3	
	IV	0	0	0	
Histological grade					0.62
	Well	4	8	12	
	Mod	0	1	1	
	Poor	0	2	2	
	Mucinous	0	1	1	
Lymphatic invasion					0.62
	+	0	2	2	
	−	4	10	14	
Vessel invasion					0.182
	+	0	4	4	
	−	4	8	12	
Neural invasion					0.712
	+	1	2	3	
	−	3	10	13	
Residual tumor					0.18
	R0	3	11	14	
	R1	1	0	1	
	R2	0	1	1	
Adjuvant chemotherapy				0.712
	+	1	2	3	
	−	3	10	13	

**Figure 9:**
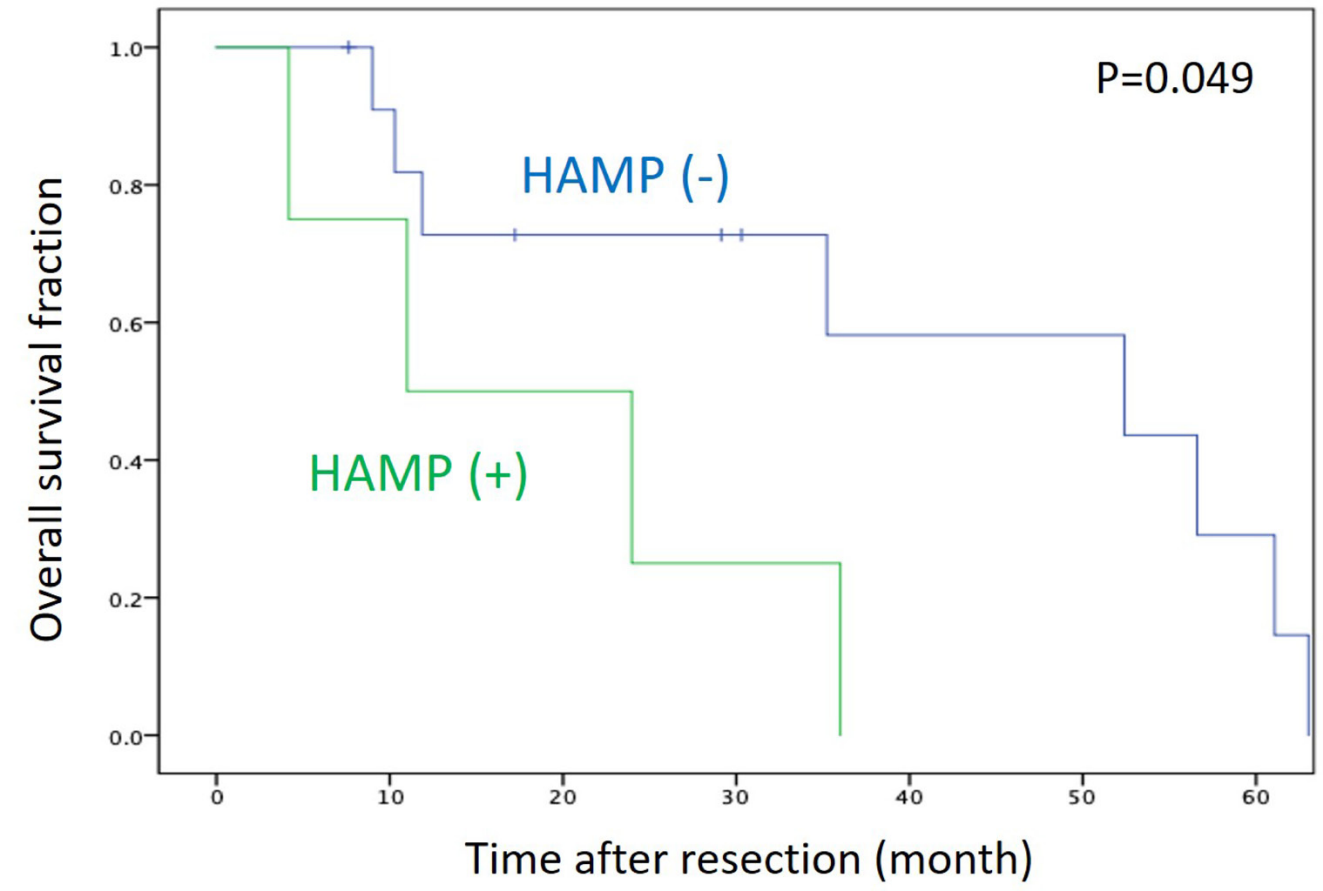
Kaplan–Meier survival curve of patients with PDAC after surgical resection. Survival of patients with HAMP+ tumors was significantly shorter those with HAMP− tumors (P = 0.049 by log-rank test).

We then compared survival between patients with HAMP+ and those with HAMP-. The overall survival was significantly shorter in patients with HAMP+ than in those with HAMP− (*P* = 0.049) (Figure 9).

Using Cox proportional hazard model, we investigated factors associated with prognosis in 16 patients with PDAC (Table 2). Univariate analysis identified HAMP+ as one of the factors predicting poor prognosis (hazard ratio, 3.69), though the association was not statistically significant (*P* = 0.066).

**Table 2:** Univariate analysis of factors predicting poor prognosis in patients with PDAC

	Univariate analysis			
	HR	95%CI		*p*
Age (>65/≤65)	0.712	0.166	3.06	0.648
Gender (M/F)	0.954	0.286	3.182	0.939
CEA	1.398	0.42	4.647	0.585
CA19-9	0.259	0.841	0.056	1.189
UICC Stage (III, IV / I, II)	0.883	0.106	7.369	0.909
Histological grade (poor/others)	0.933	0.194	4.498	0.932
Lymphatic invasion (+/-)	2.619	0.315	21.742	0.373
Vessel invasion (+/-)	2.402	0.51	11.319	0.268
Neural invasion (+/-)	2.222	0.467	10.565	0.316
Residual tumor (+/-)	2.558	0.283	23.091	0.403
Adjuvant chemotherapy (+/-)	0.827	0.096	7.156	0.863
HAMP (+/-)	3.693	0.919	14.842	0.066

## DISCUSSION

In the present study, we investigated expression profiling of genes involved in HA metabolism in a panel of PDAC cell lines and tissues. The following findings were obtained. First, expression profiling identified HAMP in a subset of PDAC cell lines and tissues. Second, HAMP+ cell lines were more susceptible to treatment with inhibitors of HA synthesis (4-MU) or degradation (dextran sulfate). Third, survival of PDAC patients with HAMP+ tumors was significantly shorter than those with HAMP- tumors. These findings suggest the presence of a novel phenotype associated with activated HA metabolism pathways and poor prognosis in PDAC.

Global genomic analyses using microarrays revealed that PDAC is characterized by frequent genetic alterations in a core set of 12 cellular signaling pathways and processes [19]. These included KRAS signaling, TGFβ signaling, JNK signaling, integrin signaling, Wnt/Notch signaling, Hedgehog signaling, control of G1-S phase transition, apoptosis, DNA damage control, small GTPase control, invasion, and hemophilic cell adhesion [19]. We demonstrate that, in addition to these pathways, HA metabolism pathway is also deregulated in PDAC. Thus, multiple signaling pathways are genetically or epigenetically altered in PDAC, which may explain its aggressive biological and clinical features.

In the present study, we demonstrated a significant positive correlation between mRNA expressions of *HAS2*, *HAS3*, *HYAL1*, and *KIAA1199*. These genes are located at different chromosomal loci, and transcriptions of these genes are independently regulated by different mechanisms. In fact, *HAS2* gene was localized to the chromosome 8q24.12, while *HAS3* gene was localized to the chromosome16q22.1 [8]. *HYAL1* gene was localized to the chromosome 3p21.3 [20], while *KIAA1199* gene was localized to the 15q25.1 [21]. Therefore, the mechanism by which transcription levels of these independent genes are simultaneously elevated is unknown. One possible mechanism is abundance of transcription factor(s) shared by these genes. For example, *HAS2* and *HAS3* have binding sites for Sp1, a transcription factor known to be overexpressed in a subset of PDAC [22], in their promoter regions [23, 24]. Another possible mechanism is aberrant hypomethylation, which has been associated with overexpression of multiple genes in PDAC [25]. In support for this idea, our previous study have shown that transcriptional expressions of *HAS2* and *HAS3* are regulated by DNA methylation in PDAC cells [26]. Finally, it is also possible that gene amplification is a mechanism underlying the overexpression of these genes. For example, frequent amplification (copy number gains) of the chromosomal loci harboring the *HAS2* gene (8q24) has been reported in PDAC [27].

In the present study, expression profiling of *HAS2*, *HYAL1*, and *KIAA1199* identified HAMP+ in 25% of PDAC patients who underwent surgery. Survival analysis revealed that patients with HAMP+ tumors showed shorter survival time than those with HAMP- tumors, though the number of patients was limited. Further studies in a larger number of patients are definitely needed to confirm the prognostic significance of HAMP in PDAC.

The present study suggests a possible personalized treatment strategy selectively for HAMP+ PDAC. First, inhibition of HA synthesis may be an ideal and straightforward treatment strategy [28]. One agent that has received increasing attention is 4-MU, which inhibits HA synthesis by acting as a competitive substrate for UDP-glucuronosyltransferase (UGT) and by downregulating HAS2 and HAS3 [29, 30]. Notably, 4-MU, also known as hymecromone, is already used in several countries as a drug to improve liver function or to treat biliary spasm without any serious side effects [31]. Previous studies have shown that 4-MU and its derivatives inhibit the growth and metastasis of PDAC *in vitro* and *in vivo* [32, 33]. Our present study showed that HAMP+ cell lines were more susceptible to 4-MU than HAMP− cell lines. Therefore, this drug should be tested selectively for PDAC patients with HAMP+ tumors in the future.

Inhibitors of HA degradation (hyaluronidase) may represent another treatment option against HAMP+ PDAC. One of the hyaluronidase inhibitors is glycyrrhizin, also known as glycyrrhizic acid, which has been shown to display anticancer properties [34]. Glycyrrhizin has also been used as a drug delivery carrier for cancer therapy [35]. We also showed that a novel hyaluronidase inhibitor, Hyaluromycin, inhibits proliferation and migration of PDAC cells [36]. These promising agents should be tested for the selective treatment of HAMP+ PDAC in preclinical and clinical studies.

In conclusion, we identified a novel phenotype, HAMP, associated with activated HA metabolism pathways in PDAC. This phenotype should be further investigated as a prognostic marker as well as a target for personalized medicine.

## MATERIALS AND METHODS

### Cell lines and reagents

We used 10 PDAC cell lines, AsPC-1, BxPC-3, Capan-2, CFPAC-1, MIAPaCa-2, PANC-1, SW1990 (American Type Culture Collection, Manassas, VA, USA), KP-2, KP-3 (JCRB Cell Bank, Osaka, Japan), and NORP-1 (RIKEN BRC Cell Bank, Tsukuba, Ibaraki, Japan). An immortalized cell line derived from human pancreatic duct, HPDE, was a kind gift from Dr. M.S. Tsao (Dept. of Pathology, Univ. of Toronto, Canada). PDAC cell lines were maintained in RPMI1640 medium (Life Technologies, Grand Island, NY, USA) supplemented with 10% fetal bovine serum (FBS) (Life Technologies) and 1% streptomycin and penicillin (Life Technologies). HPDE was maintained in HuMedia-KG2 (KURABO, Osaka, Japan), in a 5% CO^2^ incubator at 37° C. 4-Methylumbelliferone (4-MU) and dextran sulfate were purchased from SIGMA-ALDRICH Corp. (St. Louis, MO, USA).

### Patients and tissue sampling

Tissue specimens were collected from 16 PDAC patients who underwent surgical resection between 2013 and 2018 in our department. This study was approved by the ethical committee of our institution (University of Occupational and Environmental Health, Kitakyushu, Japan), and written informed consent was obtained from all patients. Frozen tissues were harvested from tumor and non-tumorous pancreata far from the tumor (confirmed under H&E staining) and minced into smaller pieces. The minced tissues were then homogenized by a homogenizer and processed for RNA extraction.

### Quantitative real-time RT-PCR

Total RNA was isolated from cell lines and homogenized tissues using RNeasy Mini Kit (QIAGEN GmbH, Hilden, Germany) according to the manufacture’s protocol. First strand cDNA was synthesized from 1.0 μg of total RNA. Real-time mRNA expression analysis of HA-related genes (*HAS2*, *HAS3*, *HYAL1*, and *KIAA1199*) and a housekeeping gene (GAPDH) for control was performed using TaqMan@ Gene Expression Assays and Step One Plus real-time PCR system (Thermo Fisher Scientific Inc., Waltham, MA, USA) according to the manufacture’s instruction. The relative quantification was given by the Ct values, determining the reactions for target genes and an internal control gene in all samples.

### Measurements of HA concentrations

Cells (1.0 × 10^5^ cells/ml) were cultured in a serum-free medium (RPMI1640 without FBS) for 48 hours and the culture medium was collected for measurements of HA concentrations. The concentration of total HA was measured using the Quantikine ELISA Hyaluronan Immunoassay (R&D Systems Inc., Minneapolis, MN, USA). Assays were triplicated and the average concentrations were determined.

### Cell migration assay

The migratory ability of cells was determined by transwell cell migration assay using cell culture inserts equipped with a filter membrane containing 8 μm pores (BD Biosciences, Franklin Lajes NJ, USA). The lower chamber was filled with RPMI1640 containing 10% FBS. The upper chamber was filled with 2.0 × 10^4^ cells in the RPMI1640 containing 1% FBS. After 24 h incubation, the cells remaining on the upper side of the filters were removed. The cells on the bottom surface of the membrane were stained with hematoxylin and eosin and the number of cells that had migrated to the bottom surface of the membrane were counted in five randomly selected microscopic fields in each sample.

### Statistical analysis

Statistical analyses were performed using SPSS statistical software version 24.0 (SPSS, Chicago, Illinois, USA). The correlation between expression of genes among PDAC cell lines was determined using Spearman’s rank correlation coefficient. Student’s t-test and Mann-Whitney *U* test were used for group comparison. Survival curves were constructed with Kaplan–Meier method and compared using the log-rank test. For univariate analysis of prognostic factors, we used Cox proportional hazard model. A *P*-value of < 0.05 was considered statistically significant.
